# Fast track program in liver resection: a PRISMA-compliant systematic review and meta-analysis

**DOI:** 10.1097/MD.0000000000004154

**Published:** 2016-07-18

**Authors:** Emad Ali Ahmed, Roberto Montalti, Daniele Nicolini, Paolo Vincenzi, Martina Coletta, Andrea Vecchi, Federico Mocchegiani, Marco Vivarelli

**Affiliations:** a Hepatobiliary and Abdominal Transplantation Surgery, Department of Experimental and Clinical Medicine, Polytechnic University of Marche, Ancona, Italy; b Hepatobiliary and Pancreatic Surgery Unit, General Surgery Department, Sohag University, Sohag, Egypt.

**Keywords:** enhanced recovery, fast track, hepatectomy, liver resection, multimodal approach, systematic review and meta-analysis

## Abstract

**Background:**

FT program (FT) is a multimodal approach used to enhance postoperative rehabilitation and accelerate recovery. It was 1st described in open heart surgery, then modified and applied successfully in colorectal surgery. FT program was described in liver resection for the 1st time in 2008. Although the program has become widely accepted, it has not yet been considered the standard of care in liver surgery.

**Objectives:**

we performed this systematic review and meta-analysis to evaluate the impact of using the FT program compared to the traditional care (TC), on the main clinical and surgical outcomes for patients who underwent elective liver resection.

**Methods:**

PubMed/Medline, Scopus, and Cochran databases were searched to identify eligible articles that compared FT with TC in elective liver resection to be included in this study. Subgroup meta-analysis between laparoscopic and open surgical approaches to liver resection was also conducted. Quality assessment was performed for all the included studies. Odds ratios (ORs) and mean differences (MDs) were considered as a summary measure of evaluating the association in this meta-analysis for dichotomous and continuous data, respectively. A 95% confidence interval (CI) was reported for both measures. *I*^2^ was used to assess the heterogeneity across studies.

**Results:**

From 2008 to 2015, 3 randomized controlled trials (RCTs) and 5 cohort studies were identified, including 394 and 416 patients in the FT and TC groups, respectively. The length of hospital stay (LoS) was markedly shortened in both the open and laparoscopic approaches within the FT program (*P* < 0.00001). The reduced LoS was accompanied by accelerated functional recovery (*P* = 0.0008) and decreased hospital costs, with no increase in readmission, morbidity, or mortality rates. Moreover, significant results were found within the FT group such as reduced operative time (*P* = 0.03), lower intensive care unit admission rate (*P* < 0.00001), early bowel opening (*P* ≤ 0.00001), and rapid normal diet restoration (*P* ≤ 0.00001).

**Conclusion:**

FT program is safe, feasible, and can be applied successfully in liver resection. Future RCTs on controversial issues such as multimodal analgesia and adherence rate are needed. Specific FT guidelines should be developed for liver resection.

## Abstract 2

Supplemental Digital Content is available in the text

## 1 Introduction

Liver resection is now the preferred treatment for a variety of benign and malignant liver diseases. Major abdominal surgical procedures such as liver resections cause a considerable surgical stress reaction and possible disturbance of metabolic functions. In addition, liver resection has its specific complications, such as postoperative hemorrhage, biliary leakage, and even liver failure.^[1]^

Fast track (FT) program, recently referred to as the enhanced recovery after surgery program, is considered one of the modern protocols used to reduce morbidity and accelerate convalescence. FT combines various approaches that are used in the care of patients who undergo elective operations. These approaches include preoperative information and education of the patients, avoiding bowel preparation and prolonged fasting, using short-acting anesthesia, keeping normal body temperature, choosing minimally invasive techniques, optimizing perioperative fluid therapy, avoiding insertion of nasogastric tubes and peritoneal drains, pain control, and aggressive postoperative rehabilitation, including early ambulation and enteral nutrition combined with nutritional supplements. The core principle of the FT program lies in the combination of these approaches, aiming for reducing the surgical stress response and organ dysfunction, thereby markedly shortening the time required for a full recovery.^[2]^

Before the introduction of the FT program, no institutions had a written or agreed perioperative care pathway. There were no specific multimodal measures to avoid prolonged perioperative fasting, nasogastric decompression, excessive use of intravenous fluids, prophylactic abdominal drains, and postoperative immobilization. The postoperative traditional care (TC) emphasized prolonged rest for both the patient and the gastrointestinal tract.^[3]^

In 1990, Krohn et al^[4]^ described the “accelerated recovery” program, which was successfully used to achieve better outcomes in patients who underwent open heart surgery. This program was later termed “fast track” by Engelman et al^[5]^ in 1994. Kehlet^[6]^ modified the program in 1997 and developed a multimodal approach to be applied to different types of interventions. Subsequently, it was applied successfully to colonic surgery in 1999.^[7]^ The program has become widely accepted, and several components of the program are increasingly being implemented in modern operative care worldwide. Despite this, FT program has not yet been accepted as the standard of care in many fields, including liver surgery. van Dam et al^[3]^ described the use of the FT program in liver resection for the 1st time in 2008.

To be implemented, the FT program requires cooperative teamwork from surgeons, anesthesiologists, physical therapists, nurses, and radiologists.^[8]^

We performed this systematic review and meta-analysis to evaluate the impact of using the FT program compared to TC, on the main clinical and surgical outcomes for patients who underwent elective liver resection.

## 2 Methods

This meta-analysis was conducted according to the preferred reporting items for systematic review and meta-analysis statement.^[9]^ Ethical approval was not necessary because this study was a systematic review and meta-analysis. Therefore, our data were based on published studies only.

### 2.1 Protocol registration

The protocol for this systematic review and meta-analysis was registered into the PROSPERO database (www.crd.york.ac.uk/prospero) with registration number CRD42015020830.

### 2.2 Eligibility criteria

Only studies that clearly compared the FT program to TC in patients who underwent elective liver resection were included in the systematic review and, if applicable, included in the meta-analysis. If more than 1 study was reported by the same institute, only the most recent or higher level study was included.

Unpublished studies, abstracts, letters, editorials, reviews without original data, and case reports were excluded. Studies that lacked a control group, compared the FT program with a non-TC pathway or compared the FT program in both arms were also excluded.

### 2.3 Search strategy

PubMed/MEDLINE, Scopus, and Cochrane databases were searched to identify all published full-text articles on the use of the FT program in liver resection. Searches were limited to English-language studies only.

The following combination of keywords were used: (“Fast Track” OR “Fast-Track” OR “Enhanced Recovery” OR “ERAS” OR “Rapid Recovery” OR “Early Recovery” OR “Early Discharge” OR “Rapid Discharge” OR “Multimodal Approach” OR “Multi-Modal Approach” OR “Multimodal Program” OR “Multi-Modal Program” OR “Multimodal Protocol” OR “Multi-Modal Protocol” OR “Multimodal Measures” OR “Multi-Modal Measures”) AND (“Liver” OR “Hepatic” OR “Hepatectomy” OR “Hepatectomies” OR “Hepatobiliary” OR “Hepato-Biliary”). For all databases, the last search was completed on May 25, 2015.

### 2.4 Study selection

Two authors (EAA, RM) independently screened the titles and abstracts of the primary studies that were identified in the electronic search. Then, the bibliographies of relevant articles were manually reviewed to identify additional trials. Duplicate studies were excluded. Discrepancies between the 2 reviewers were resolved by a 3rd expert reviewer (MV).

### 2.5 Data extraction and outcomes of interest

The following parameters were extracted from each study by 2 authors (EAA, DN) independently: name of the first author and his/her affiliation, year of publication, study design, the number of patients in each arm, patient characteristics, and study quality.

All relevant texts, tables, and figures were reviewed for data extraction; whenever further information was required, the corresponding author of the paper was contacted by e-mail.

To evaluate the impact of the FT program in liver resection compared to the TC, the following outcomes were studied: operative time, blood loss, the need for blood transfusion, and conversion rate in the laparoscopic resection. In addition, postoperative events were studied such as length of hospital stay (LoS), functional recovery, intensive care unit (ICU) admission, 1st bowel opening, restoration of oral fluid and normal diet, morbidity and mortality rates, pain score, C-reactive protein (CRP) level, hospital cost, readmission rate, and quality of life (QoL). The primary outcomes of this analysis were postoperative LoS and functional recovery, whereas the others were considered secondary outcomes.

Hospital stay was defined as the interval from the day of surgery to the day of actual discharge from the hospital. Functional recovery was determined by the number of days to reach a certain criteria of recovery, which included good pain control with oral analgesia, tolerance of solid food, normal body temperature, independent mobilization, normal or decreasing the serum bilirubin level, and willingness of the patients to be discharged. Readmission was defined as any hospital readmission within 30 days after discharge.

Operative time was defined as the interval from the incision to the suturing of the skin. The 1st bowel opening was determined by the 1st passage from the bowel of either stool or flatus while pain score was evaluated by using a visual analog scale (VAS) ranging from 0 to 10. Postoperative mortality was defined as the occurrence of death during hospitalization or within 30 days after surgery, while postoperative morbidity included the complication rate from the time of surgery to 90 days after discharge.

For assessment of the QoL, European quality of life-five dimensions model (EQ-5D)^[10]^ was used. Repeated EQ-5D measurements were obtained in the postoperative period, and differences between groups in health-related QoL were calculated by using the area under the curve method. Alternatively, QoL could be assessed by completing the general comfort questionnaire (GCQ) based on the Kolcaba comfort line^[11]^ which is determined on hospital discharge.

### 2.6 Qualitative analysis of each study

The quality of the retrospective studies were assessed according to the Newcastle–Ottawa Scale that scores patient selection, comparability between the 2 study groups, and assessment of outcomes. Studies that achieved ≥7 points were defined as “good” quality. The quality of the randomized controlled trials (RCTs) was assessed according to the Cochrane Collaboration tool for assessing risk of bias.^[12]^ This tool analyzes the following criteria: random sequence generation, allocation concealment, blinding of participants and personnel, blinding of outcome assessment, incomplete outcome data, selective reporting, and other bias. For each entry based on the risk of bias assessment guidelines, we made a judgment (low risk of bias, high risk of bias, or uncertain). All disagreements were resolved by discussion until a consensus agreement was achieved.

### 2.7 Statistical analysis

The meta-analysis was performed by using Review Manager (“RevMan” [Computer program], Version 5.3. Copenhagen: The Nordic Cochrane Centre, the Cochrane Collaboration, 2014). Odds ratios (ORs) with Mantel–Haenszel method were used as a summary measure of efficacy for dichotomous data while mean differences (MDs) with inverse variance method were applied for continuous variables. A 95% confidence interval (CI) was reported for both measures. If the study provided medians and ranges instead of means and standard deviations, the means and standard deviations were imputed, as described by Hozo et al.^[13]^ The fixed-effect model was used when no heterogeneity was detected among studies, while the random-effects model was preferred when variance existed. Statistical heterogeneity was evaluated by using the *I*^2^ statistic. *I*^2^ values of 0% to 25%, 25% to 50%, and >50% were considered indicative of homogeneity, moderate heterogeneity, and high heterogeneity, respectively. Forest plots were constructed and all data were considered statistically significant for *P* ≤ 0.05.

In this meta-analysis, we pooled the results from all the included studies in regards to the primary and secondary outcomes. In addition, we meta-analyzed the RCTs separately for each outcome; the results were only discussed if a difference was found compared to the pooled meta-analysis.

### 2.8 Risk of bias across studies

Assessment of the risk of publication bias across series for all outcome measures will be conducted according to the guidelines established by the Cochrane Handbook for Systematic Reviews.^[14]^

### 2.9 Additional analyses

Due to the variability of surgical approaches to liver resection (by either laparoscopy or open surgery), we will include a meta-analysis of subgroups for outcomes affected by this technical difference, such as operative outcomes, postoperative LoS, and morbidity and mortality rates.

## 3 Results

### 3.1 Eligible studies

**Figure 1:**
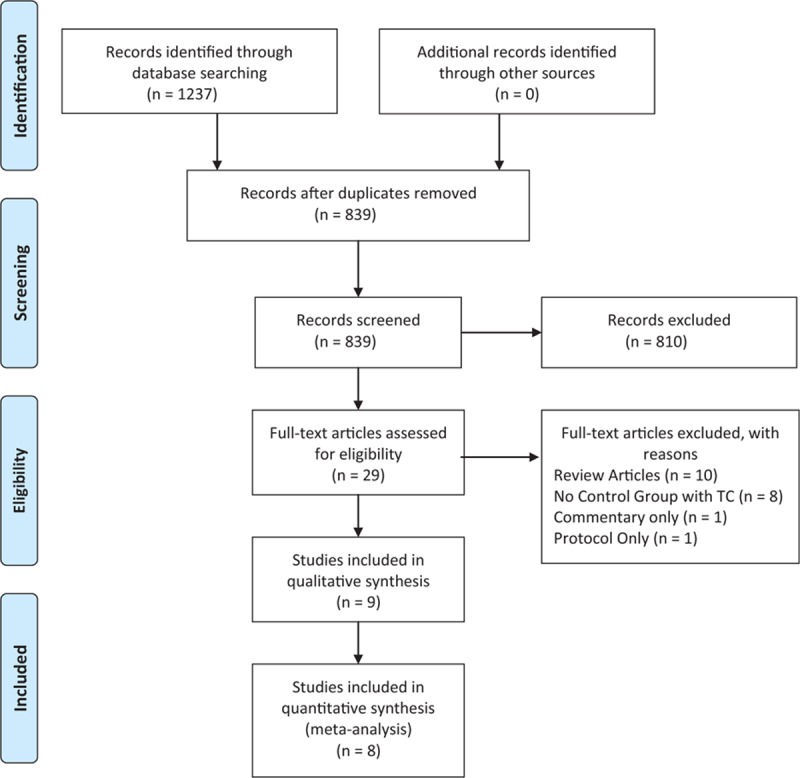
Preferred reporting items for systematic review and meta-analysis (PRISMA) flow diagram.

The preferred reporting items for systematic review and meta-analysis flow diagram of the systematic literature search are shown in Fig. 1. The literature search included all articles published up to May 25, 2015, which yielded 1237 articles. After removing duplicates, the titles and abstracts of 839 articles were reviewed. Of these, 830 articles were excluded for the following reasons: 810 were not related to the FT program in liver resection, 10 were review articles,^[1,15–23]^ 8 did not include a TC control group,^[24–31]^ 1 was a commentary,^[32]^ and 1 was only a protocol description.^[33]^

Nine comparative studies published between 2008 and 2015 matched our inclusion and exclusion criteria and were selected for inclusion in our systematic review and meta-analysis.^[3,8,34–40]^ The abstract for one of the articles was available but the full-text could not be found in any database, despite after 6 weeks of dedicated searching. The corresponding author of the article was contacted through e-mail but we did not receive a response.^[37]^ Therefore, a final total of 8 studies was included in the quantitative synthesis.

### 3.2 Study characteristics

The characteristics of these studies are summarized in Table 1. The 8 studies included 810 patients: 394 in the FT group and 416 in the TC group. The sample size of these studies ranged from 26 to 161 patients. The patient characteristics, including sex, age, and American society of anesthesiologists score, were collected from all of the included studies.

**Table 1:**
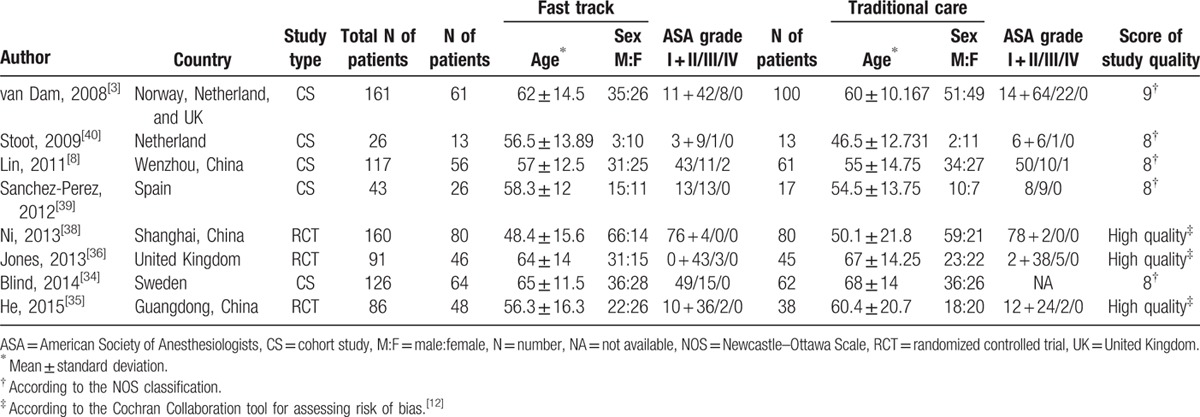
Characteristics of the included studies.

The indications and extent of liver resection are shown in Table, Supplemental digital Content 1. A detailed FT program, including the most frequently described elements in each study and the respective adherence rate (if reported), is summarized in Table 2.

**Table 2:**
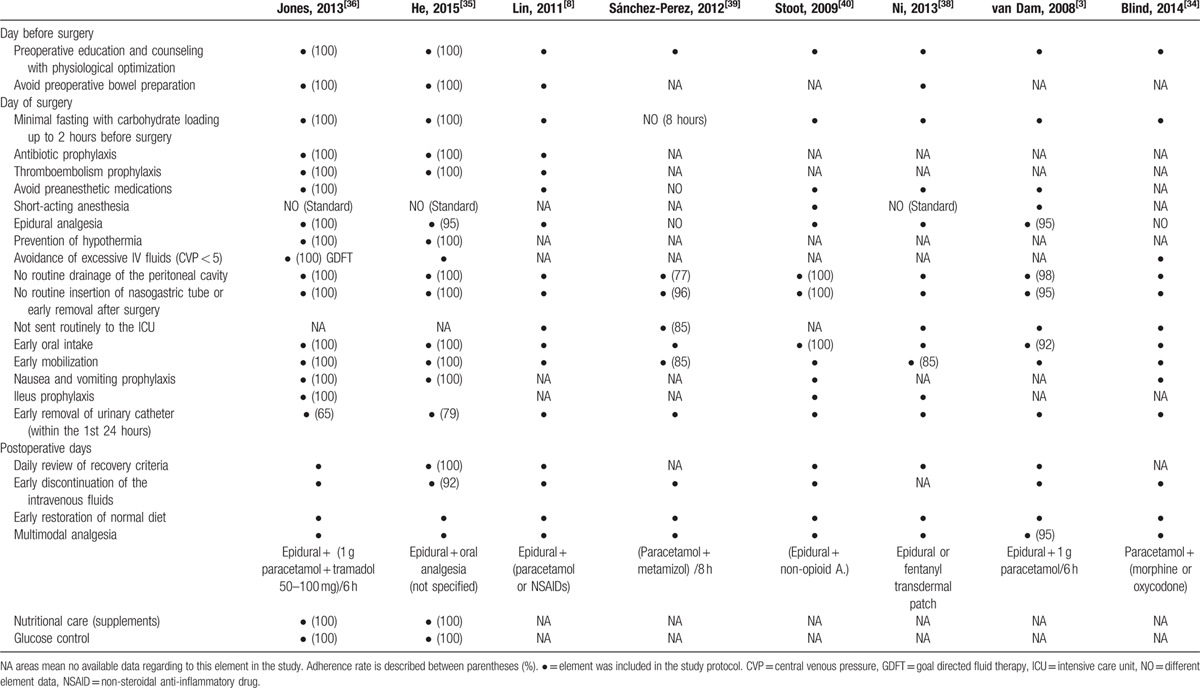
Fast track program in liver resection with adherence rate in each study (if reported).

The final selected articles included 3 RCTs^[35,36,38]^ and 5 cohort studies.^[3,8,34,39,40]^ Three studies were conducted by laparoscopy^[35,39,40]^ and 5 by open surgery.^[3,8,34,36,38]^

### 3.3 Quality assessment

**Figure 2:**
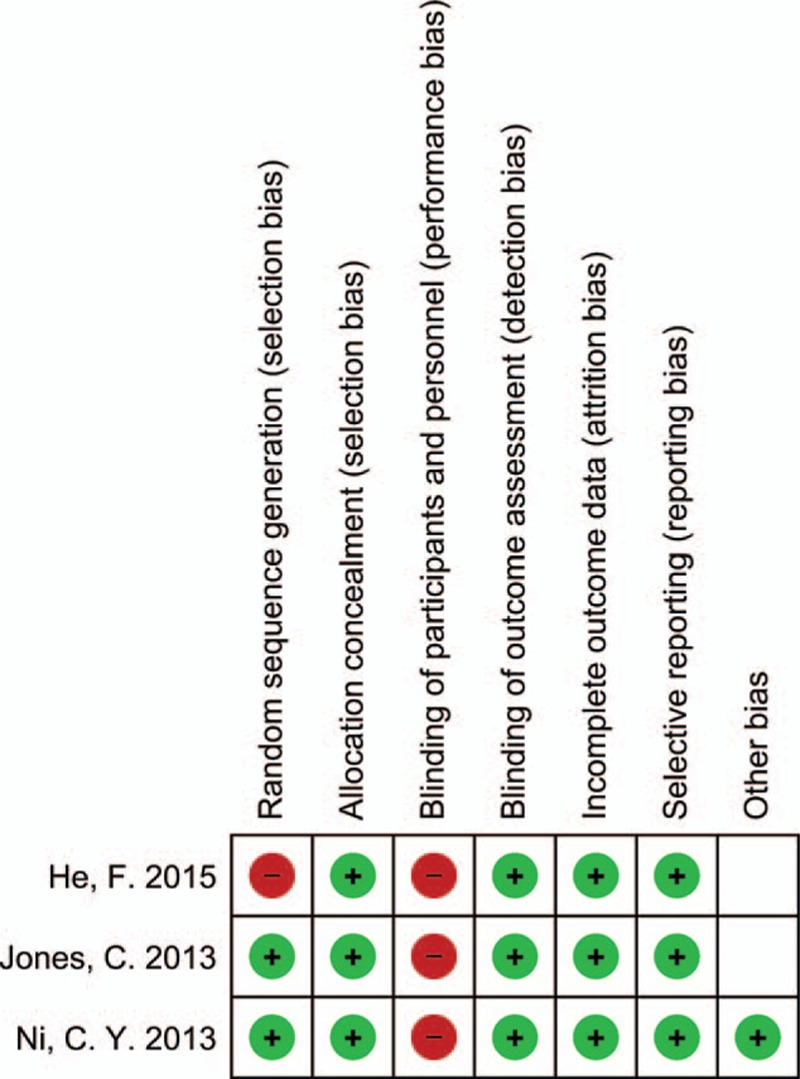
Risk of bias summary in randomized controlled trials (RCTs). The symbol of (−) indicates that there is a high risk of bias, of (+) indicates a low risk of bias and the blank field indicates uncertainty.

All included RCTs are of high quality as summarized in Fig. 2. One cohort study was scored 9,^[3]^ while the remaining 4 cohort studies were scored 8.^[8,34,39,40]^ Consequently, the quality of these studies was considered good, according to the Newcastle–Ottawa Scale.

### 3.4 Meta-analysis of the primary outcomes

#### 3.4.1 Length of hospital stay (LoS)

**Figure 3:**
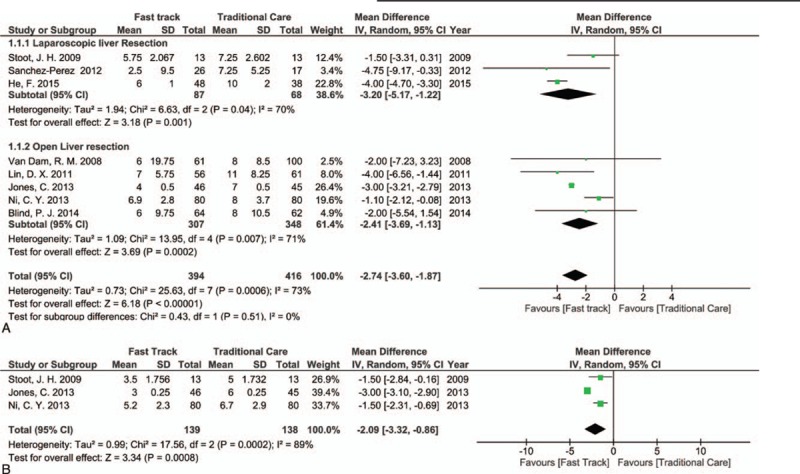
Forest plot of comparison: fast track versus traditional care, outcome: (A) length of hospital stay; (B) functional recovery.

Hospital stay was reported in 5 studies of open liver resection,^[3,8,34,36,38]^ and a highly statistically significant result between the FT and TC groups was found, in favor of the FT group (MD [CI 95%] = −2.41 [−3.69, −1.13]; *P* = 0.0002), with marked heterogeneity (*I*^2^ = 71%). LoS was also reported in all studies of laparoscopic liver resection,^[35,39,40]^ and the findings demonstrated a significant result in favor of the FT group (MD [CI 95%] = −3.20 [−5.17, −1.22]; *P* = 0.001), with marked heterogeneity (*I*^2^ = 70%). Overall, these results demonstrated a highly statistically significant difference between the FT and TC groups, in favor of the FT group (MD [CI 95%] = −2.74 [−3.60, −1.87]; *P* < 0.00001). However, marked heterogeneity between these studies was observed; *I*^2^ = 73% (Fig. 3A).

#### 3.4.2 Functional recovery

Three studies, including 2 RCTs and 1 cohort study, reported data on functional recovery^[36,38,40]^ and showed statistically significant results in favor of the FT group (MD [CI 95%] = −2.09 [−3.32, −0.86]; *P* = .0008, *I*^2^ = 89%, Fig. 3B).

### 3.5 Meta-analysis of the secondary outcomes

#### 3.5.1 Operative time (minute)

**Figure 4:**
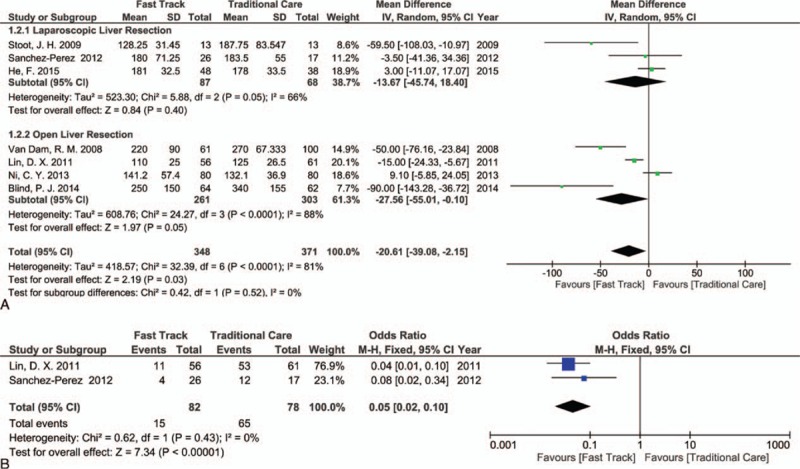
Forest plot of comparison: fast track versus traditional care, outcome: (A) operative time; (B) intensive care unit (ICU) admission rate.

In regards to open liver resection, 4 studies reported the data for the operative time^[3,8,34,38]^ and the difference between the FT and TC groups was significant, in favor of the FT group (MD [CI 95%] = −27.56 [−55.01, −0.10]; *P* = 0.05), with marked heterogeneity (*I*^2^ = 88%). On the other hand, 3 studies of laparoscopic liver resection reported the data for the operative time^[35,39,40]^ and showed that the difference between the FT and TC groups was not statistically significant (MD [CI 95%] = −13.67 [−45.74, 18.40]; *P* = 0.40), with *I*^2^ = 66%. Overall, the differences in outcome were significant statistically in favor of the FT group (MD [CI 95%] = −20.61 [−39.08, −2.15]; *P* = 0.03), with marked heterogeneity between studies; *I*^2^ = 81% (Fig. 4A).

#### 3.5.2 Blood loss (mL)

Studies that reported the intraoperative blood loss during open^[3,8,34,36,38]^ or laparoscopic^[35,40]^ hepatectomy did not show a statistically significant difference between the FT and TC groups (MD [CI 95%] = −74.67 [−152.50, 3.17]; *P* = .06, *I*^2^ = 68%, see Figure, Supplemental Digital Content 2).

#### 3.5.3 Need for blood transfusion

Three studies of open liver resection^[8,36,38]^ and 3 studies of laparoscopic liver resection^[35,39,40]^ reported the need for blood transfusion data and showed not statistically significant results (OR [CI 95%] = 1.03 [0.67, 1.60]; *P* = .89, *I*^2^ = 1%, see Figure, Supplemental Digital Content 3).

#### 3.5.4 Conversion rate

The conversion rate from laparoscopic into open liver resection was reported in all studies of laparoscopic liver resection^[35,39,40]^ and showed no statistically significant differences between both groups (OR [CI 95%] = 0.99 [0.34, 2.87]; *P* = .099, *I*^2^ = 0%, see Figure, Supplemental Digital Content 4).

#### 3.5.5 ICU admission and stay

Three studies reported the data regarding ICU admission and stay;^[8,38,39]^ 2 reported the percentages of ICU admission of patients,^[8,39]^ and there were fewer admitted FT group patients with high statistical difference than TC group patients, (OR [CI 95%] = 0.05 [0.02, 0.10]; *P* < 0.00001, *I*^2^ = 0%, Fig. 4B). The 3rd study^[38]^ described the length of postoperative ICU stay, which showed a trend toward decreased length of ICU stay in the FT group, but this was not statistically significant (1.2 ± 0.2 vs 1.3 ± 0.6 days; *P* = 0.08).

#### 3.5.6 First bowel opening

**Figure 5:**
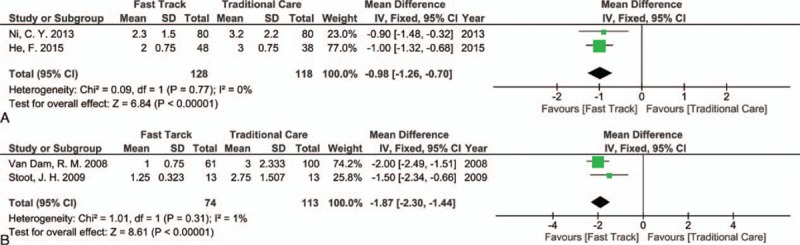
Forest plot of comparison: fast track versus traditional care, outcome: (A) first bowel opening; (B) restoration of normal diet.

Two RCTs described this outcome^[35,38]^ and showed that differences in the results between the FT and TC groups were statistically significant, where there was earlier bowel activity in the FT group (MD [CI 95%] = −0.98 [−1.26, −0.70]; *P* < 0.00001, *I*^2^ = 0%, Fig. 5A).

Jones et al^[36]^ reported that bowel sounds recurred sooner (*P* < 0.001) and flatus passed earlier (*P* = 0.008) in the FT group, but these data were not included in the meta-analysis because there were, only the *P* values.

#### 3.5.7 Restoration of oral fluid and normal diet

Stoot et al^[40]^ reported that in nearly all patients in both the FT and TC groups, oral fluid intake was resumed within the 1st 24 hours after surgery (1 ± 0.577 vs 2 ± 1.826, respectively), and there was no statistically significant difference between the 2 groups (*P* = 0.86). Jones et al^[36]^ reported that patients in the FT group resumed oral intake earlier after surgery than those in the TC group (median 115 vs 330 minutes; *P* < 0.001, respectively) and drank more fluids in the 1st 24 hours (1375 vs 810 mL; *P* < 0.001, respectively). However, these data had no range, so it was not possible to include them in our meta-analysis. van Dam et al^[3]^ also described that fluid intake was resumed 4 hours after surgery in 56 patients (92%) in the FT group.

In regards to the restoration of a normal diet, 2 studies reported these data^[3,40]^ and showed a highly significant difference between the FT and TC groups, in favor of the FT group (MD [CI 95%] = −1.87 [−2.30, −1.44]; *P* < 0.00001, *I*^2^ = 1%, Fig. 5B).

#### 3.5.8 Morbidity and mortality rates

**Figure 6:**
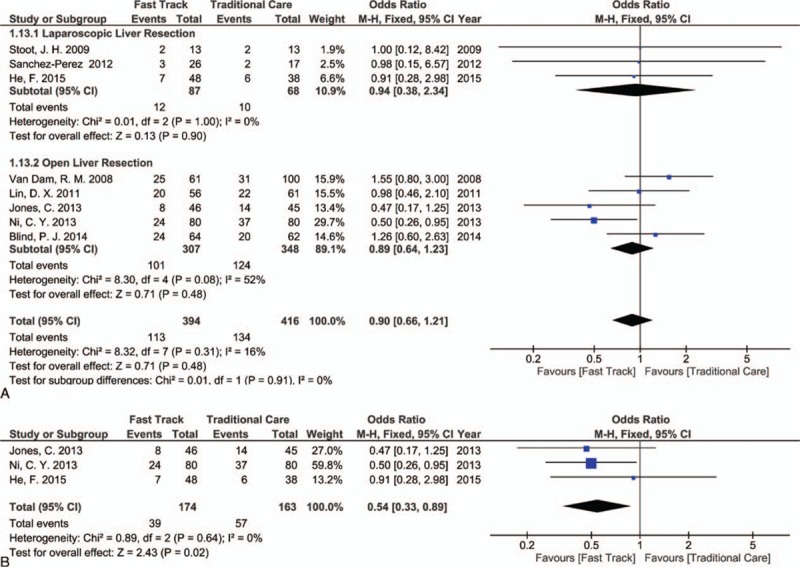
Forest plot of comparison: fast track versus traditional care, outcome: (A) morbidity rate (all studies); (B) morbidity rate (randomized controlled trials [RCTs] only).

All of the selected studies reported these outcomes.^[3,8,34–36,38–40]^ No statistically significant differences in morbidity were found between the FT and TC groups in open or laparoscopic liver resection (OR [CI 95%] = 0.90 [0.66, 1.21]; *P* = 0.48, *I*^2^ = 16%, Fig. 6A). When this meta-analysis included only RCTs,^[35,36,38]^ a significant decrease in morbidity rate was observed in the FT group (OR [CI 95%] = 0.54 [0.33, 0.89]; *P* = 0.02, *I*^2^ = 0%, Fig. 6B). No mortality was registered in the FT and TC groups after laparoscopic liver resection, and results were not significantly different between the 2 groups after open liver resection (OR [CI 95%] = 0.68 [0.14, 3.34]; *P* = 0.64, *I*^2^ = 0%, see Figure, Supplemental Digital Content 5).

#### 3.5.9 Pain score

One RCT reported data for the pain score,^[36]^ and it showed that there were no differences between the FT and TC groups except on the 2nd postoperative day (POD), where VAS was significantly lower in the FT than the TC group (2.5 ± 1.4 vs 3.3 ± 2.0, respectively; *P* = 0.04).

#### 3.5.10 C-reactive protein level (CRP)

One RCT^[38]^ reported the serum CRP level on POD 1, 3, and 5; its level in the FT group was significantly lower than the TC group (all *P* < 0.05).

#### 3.5.11 Hospital cost

Two studies reported data for hospital costs; He et al^[35]^ showed that the average hospital costs were 9470 ± 1540 Renminbi (Chinese yuan) (RMB) between the TC group and only 7742 ± 1200 RMB in the FT group (*P* = 0.03). Lin et al^[8]^ reported that the median of charges (without range) during hospital stay was 21,004 RMB for the FT group, which is significantly less than the 26,626 RMB for the TC group (*P* < 0.05). Sanchez-Perez et al^[39]^ reported that the use of the FT program reduced hospital costs by about 30%; the study did not include any other cumulative data that could be used in our meta-analysis.

#### 3.5.12 Readmission rate

Seven studies reported the readmission rate^[3,8,34–36,39,40]^ and showed that there were no statistically significant results between the FT and TC groups (OR [CI 95%] = 1.17 [0.61, 2.23]; *P* = 0.64, *I*^2^ = 0%, see Figure, Supplemental Digital Content 6).

#### 3.5.13 Quality of life

The 3 RCTs reported this outcome; 2 of them^[35,36]^ reported it according to EQ-5D and described significant changes in QoL scores for the FT group over time. In both the FT and TC groups, the QoL measures after surgery showed an initial decrease from baseline. Over the 1st month after surgery, QoL improved considerably in the FT group. The median area under the curve was higher in the FT group than the TC group, as reported by He et al (36.9 vs 35.2; *P* = 0.04, respectively)^[35]^ and Jones et al (37.2 vs 35.6, *P* = 0.002, respectively).^[36]^

The 3rd trial^[38]^ described QoL according to GCQ and found that the mean of its measures in the FT group was higher than the TC group (101.2 ± 13.0 vs 93.4 ± 21.4, respectively; *P* < 0.01), which is statistically significant in favor of the FT group.

### 3.6 Risk of bias across studies

According to the Cochran handbook for systematic reviews, the test for publication bias is unreliable when less than 10 studies are included in a meta-analysis. Therefore, funnel plots were not constructed in this study.

## 4 Discussion

Major surgery is still associated with many undesirable consequences such as pain, cardiopulmonary, gastrointestinal, infectious, and thromboembolic complications associated with a prolonged convalescent period. The key pathogenic factor in postoperative morbidities, excluding failures of surgical and anesthetic techniques, is the surgical stress response and the subsequent increased demand for organ function, especially if it is associated with preoperative risk factors and comorbidities.^[4,6]^

FT program aims to reduce morbidity and accelerate convalescence through preoperative information and education of the patients, stress reduction, optimizing pain control, and aggressive postoperative rehabilitation, including early enteral nutrition and ambulation associated with nutritional supplements.^[2,6]^

Stress reduction can be obtained by avoiding preoperative bowel preparation and prolonged fasting; patients were allowed to eat and drink until 6 hours, with clear fluids permitted until 2 hours before surgery.^[3,30]^ Using short-acting anesthesia and regional anesthesia and analgesia, avoiding hypothermia, and choosing minimally invasive techniques were essentials. In particular, some authors suggested that laparoscopic liver resection could improve postoperative recovery, decrease morbidity, and therefore, may enhance the results of FT program.^[17,41,42]^ Goal-directed fluid therapy based on individual optimization of stroke volume has been demonstrated to improve outcomes in liver surgery through keeping lower central venous pressure (CVP) during the resection procedure and enhancing gastrointestinal and pulmonary function postoperatively.^[36,43–45]^ Routine drainage of the peritoneal cavity and insertion of nasogastric tube are not recommended within the FT program.^[3]^

Optimal management of postoperative pain is a prerequisite for FT surgery and it should be a balanced multimodal analgesia.^[3,6,8,36]^ This reduces postoperative nausea and vomiting and allows for early ambulation that is recommended 8 hours after surgery or maximum delayed to the 1st POD.^[2]^ It also facilitates early enteral nutrition; patients within the FT program are encouraged to drink oral fluids 4 to 6 hours after surgery and eat normal food on the 1st POD.^[3,34,35]^

However, Anders^[46]^ pointed out potential problems with the FT program and suggested that there were adverse effects on the quality of care. He also claimed that hospitals wanted to discharge patients more quickly despite their poorer health state, to decrease their expenses. Moreover, due to the complexity of the FT program and its multiple elements, many barriers to the implementation of the program have arisen, including institutional barriers such as lack of experienced medical and nursing staff. In addition, there were intervention-specific barriers due to limited or weak supporting evidence for several elements of the program. Individual barriers arose from perioperative teams who resisted the change of traditional methods, and poor collaboration between team members.^[47,48]^ Therefore, TC still prevails in many centers as the preferred choice of many surgeons.^[49]^

This argument about the FT program resulted in a few published reports that compared the FT and TC programs, particularly in liver resection. The systematic search found 6 previous systematic reviews,^[1,16,18–20,23]^ 2 of them with meta-analyses^[16,20]^ and 3 discussed upper abdominal surgeries, including liver resections.^[18,19,23]^ None of these reviews investigated the effect of a laparoscopic approach compared with the open one within the FT program in liver resection. Moreover, none of the publications assessed the QoL, pain score, and ICU admission rate after liver resection in regards to the FT program. In addition, insufficient data about functional recovery, hospital costs, and assessment of protocol adherence were reported. In our present study, we discussed 16 outcomes and aimed to properly assess the FT program compared to the TC program in liver resection.

A meta-analysis, as a quantitative method for therapeutic evaluation, may be used when controversy persists in order to clarify the results of different studies by developing supporting evidence and recommendations. It was possible to include 8 studies in this meta-analysis, which in total contained 810 patients; 3 studies were RCTs and 5 were cohort studies.

In regards to the postoperative outcomes in this meta-analysis, LoS, which is a primary outcome was markedly shortened in both open and laparoscopic liver resections within the FT program. LoS ranged from 2.5 to 7 days compared with the TC group, which ranged from 7.25 to 11 days (*P* < 0.00001). Moreover, functional recovery was accelerated by the program (*P* = 0.0008). Currently, functional recovery is considered to be more accurate than LoS in describing the actual time needed for complete recovery, as it is not affected by other social or psychological events.^[1]^ Despite its importance, it was reported by only 3 of the included studies.^[36,38,40]^

The reduced LoS and accelerated functional recovery in the FT group are accompanied by a lower ICU admission rate (*P* < 0.00001) and markedly reduced hospital costs, with no increase in the readmission, morbidity, or mortality rates. When including only RCTs in the meta-analysis, a significant decrease in morbidity rate was noticed in the FT group (*P* = 0.02), in concordance with the results of 2 previous meta-analyses.^[16,20]^ Decreased perioperative fasting periods, restricted fluid infusion, and hypothermia prevention could be the causes of these advantages in the FT group. Maintaining perioperative normal blood glucose level prevents the sense of thirst, hunger, and anxiety.^[50]^ In addition, restricting over-hydration prevents delayed gastrointestinal function and reduces interstitial edema, lung compliance, and cardiac overload.^[35]^ Therefore, amelioration of the overall surgical stress response was resulted, associated with a successive decrease in the complication rate and enhanced recovery.

A significant result in favor of the FT group in regards to earlier bowel opening (*P* < .00001) was observed. Oral fluid intake was resumed within the 1st 24 hours after surgery nearly in all patients of the FT group, but the results were not statistically significant between the FT and TC groups. In the FT program, normal diet was restored successfully on the 1st POD compared to the TC group (*P* < 0.00001). These results may be due to the restriction of perioperative over-hydration, early ambulation, and good pain control.

Only 1 study reported the serum level of CRP, which is used as a laboratory indicator of the surgical stress response. The study showed that levels of CRP were significantly lower in the FT group compared to the TC group (*P* < 0.05).^[38]^ Also, a 1 study reported that there was no difference in pain score between the FT and TC groups, except on the 2nd POD where the VAS score was significantly lower in the FT group (*P* = 0.044).^[36]^

QoL measures are an important factor in the evaluation of these programs. After surgery, EQ-5D measures showed an initial decrease from baseline in the FT and TC groups. Over the 1st postoperative month, QoL considerably improved in the FT group.^[35,36]^ Ni et al^[38]^ assessed QoL by using a GCQ based on Kolcaba comfort line and found significantly better results in the FT group (*P* < 0.01).

In regards to the operative outcomes in this meta-analysis, surgical time was significantly reduced in patients that underwent open hepatectomy within the FT program compared to the TC group (*P* = 0.05). However, no significant difference was noticed between the 2 groups in patients that underwent laparoscopic liver resection. This effect in open hepatectomy with FT may be attributed to the differences in anesthetic agents administered, which may be associated with better hemodynamic stability and less use of vasopressors. This beneficial effect in laparoscopic hepatectomy is mitigated by the technical difficulty of the intervention, which is often time-consuming. Other operative parameters including intraoperative blood loss, blood transfusion, and conversion rate were not significantly different between the FT and TC groups.

Interestingly, it was noticed that the FT program did not add any significant advantage to patients who underwent laparoscopic liver resection; perhaps, the minimally invasive approach itself enhanced the postoperative recovery, and the introduction of the FT program in this subgroup of patients does not add any other beneficial effects.

As evidenced by a Cochrane meta-analysis,^[51]^ simply using the FT program does not guarantee improved results unless there is stringent oversight of protocol adherence by all members of the team.^[18]^ In the selected studies, there were confusing and insufficient data about the adherence to the FT program elements, and there was a high discrepancy between these elements. Only 2 out of 8 studies properly described the adherence rate to every single element of the program with a reported high compliance, except for the early removal of the urinary catheter (79% and 65% adherence rates, respectively).^[35,36]^ In addition, most of these described elements were derived from the colorectal FT program, which may be not suitable for liver surgery.

Moreover, the available data about the anesthetic agents used were heterogeneous; some of these studies proposed the standard anesthetic protocol for both groups based on opioids,^[35,36,38]^ but others used short-term anesthetic agents.^[3,40]^ The majority of the included studies considered thoracic epidural as the regional anesthesia of choice in the FT program. On the other hand, some authors considered that epidural use is recommended in colorectal surgery, but its use is questionable in liver resection, as it may impair the postoperative recovery and it should be replaced by intrathecal morphine.^[16,50,52,53]^ Furthermore, Hughes et al^[16]^ reported that a small liver remnant after major resection may be a contraindication to the administration of paracetamol as it may induce liver damage. However, recent evidence recommended that paracetamol is safe in hepatic patients when its maximum dose is reduced to 2 to 3 g/day; nonsteroidal antiinflammatory drugs and opioids are better avoided.^[54,55]^ As pain control is considered one of the most important elements in the FT program, further evaluation of analgesia with alternative methods in liver surgery is required to establish optimal results.

The nonuse of abdominal drains following liver resection is also a matter of debate. Some trials revealed that routine abdominal drainage is unnecessary after elective hepatectomy.^[56,57]^ On the other hand, other reports described many valuable diagnostic and therapeutic benefits of the drain, especially after major liver resection.^[58,59]^ As the FT program discourages the use of abdominal drains, this element should be revised within the FT program in the context of liver surgery. Perhaps, a protocol for early versus late drain removal or even no drain placement, only in minor liver resection would be applicable.

Now, well-established FT program with guidelines was developed in different kinds of surgery, including colorectal/pelvic^[60,61]^ and gynecologic/oncology surgeries,^[62]^ and in pancreaticoduodenectomy,^[63]^ cystectomy,^[64]^ and gastrectomy^[65]^ procedures. No guidelines for the implementation of the FT program in liver surgery were identified. As liver surgery is a special entity, we advise the development of well-determined FT program elements specifically for these types of operations to be used as guidelines. This allows for better evaluation and standardization of the FT program in the field of liver surgery.

Finally, this meta-analysis showed significant results in favor of the FT program in regards to LoS, functional recovery, operative time, ICU admission rate, 1st bowel opening, and restoration of a normal diet. No significant differences emerged between the FT program and TC when comparing blood loss, need for blood transfusion and conversion, readmission, and mortality rates. When considering RCTs only, significantly lower morbidity rates were observed in the FT program. None of the analyzed outcomes showed superior results in favor of the TC group.

This meta-analysis had several limitations: the majority of the included studies are retrospective in nature and there was a marked heterogeneity between the investigated outcomes. We used the random-effects model as appropriate, but this bias was impossible to overcome. Moreover, there is a marked insufficiency of available data in the literature in regards to the FT program in liver resection, and the comparison of several outcomes such as pain score with multimodal analgesia, hospital costs, the serum level of CRP, duration to 1st bowel opening, and restoration of oral fluid and normal diet. We recommend considering these outcomes in prospective randomized studies so that better judgments can be made for the FT program in liver resection.

## 5 Conclusion

LoS was markedly reduced and functional recovery was accelerated when the FT program was implemented, without increasing readmission, morbidity, or mortality rates. Based on our systematic review and meta-analysis, the FT program is safe, feasible, and can be applied successfully in liver resection. Future RCTs focusing on debated issues such as multimodal analgesia and adherence rate are needed. The development of specific FT guidelines in the field of liver resection is strongly recommended.

## Supplementary Material

Supplemental Digital Content
