# Supplementary material for: Fast track program in liver resection: a PRISMA-compliant systematic review and meta-analysis
Source: Medicine (Baltimore). 2016 Jul 18;95(28):e4154. doi: 10.1097/MD.0000000000004154 (PMC4956800; doi:10.1097/MD.0000000000004154)
Supplement: Supplemental Digital Content [file medi-95-e4154-s001.pdf]

Supplemental Digital Content

Supplemental Digital Content 1, Table that illustrates the indications and extent of liver resection of the included studies.

Supplemental Digital Content 2, Figure that illustrates the Forest plot of comparison: Fast Track vs. Traditional Care, outcome: blood loss.

	Fast track			Traditional Care				Mean Difference	
Study or Subgroup	Mean	SD	Total	Mean	SD	Total	Weight	IV, Random, 95% CI	Year
**1.3.1 Laparoscopic liver resection**									
Stoot, J. H. 2009	87.5	48.412	13	337.5	222.322	13	15.3%	-250.00 [-373.69, -126.31]	2009
He, F. 2015	350	170	48	338	190	38	20.0%	12.00 [-65.22, 89.22]	2015
**Subtotal (95% CI)**			**61**			**51**	**35.2%**	**-114.36 [-370.96, 142.23]**	
Heterogeneity: Tau² = 31554.77; Chi² = 12.40, df = 1 (P = 0.0004); I² = 92%									
Test for overall effect: Z = 0.87 (P = 0.38)									
**1.3.2 Open Liver Resection**									
Van Dam, R. M. 2008	750	1,250	61	800	1,000	100	3.8%	-50.00 [-419.88, 319.88]	2008
Lin, D. X. 2011	760	525	56	850	575	61	9.4%	-90.00 [-289.32, 109.32]	2011
Jones, C. 2013	350	181.5	46	340	123.75	45	21.3%	10.00 [-53.70, 73.70]	2013
Ni, C. Y. 2013	313.4	223.9	80	358.2	311.7	80	19.3%	-44.80 [-128.90, 39.30]	2013
Blind, P. J. 2014	300	612.5	64	500	362.5	62	11.0%	-200.00 [-375.10, -24.90]	2014
**Subtotal (95% CI)**			**307**			**348**	**64.8%**	**-43.90 [-109.42, 21.63]**	
Heterogeneity: Tau² = 1548.59; Chi² = 5.61, df = 4 (P = 0.23); I² = 29%									
Test for overall effect: Z = 1.31 (P = 0.19)									
**Total (95% CI)**			**368**			**399**	**100.0%**	**-74.67 [-152.50, 3.17]**	
Heterogeneity: Tau² = 6348.97; Chi² = 18.65, df = 6 (P = 0.005); I² = 68%									
Test for overall effect: Z = 1.88 (P = 0.06)									
Test for subgroup differences: Chi² = 0.27, df = 1 (P = 0.60), I² = 0%									

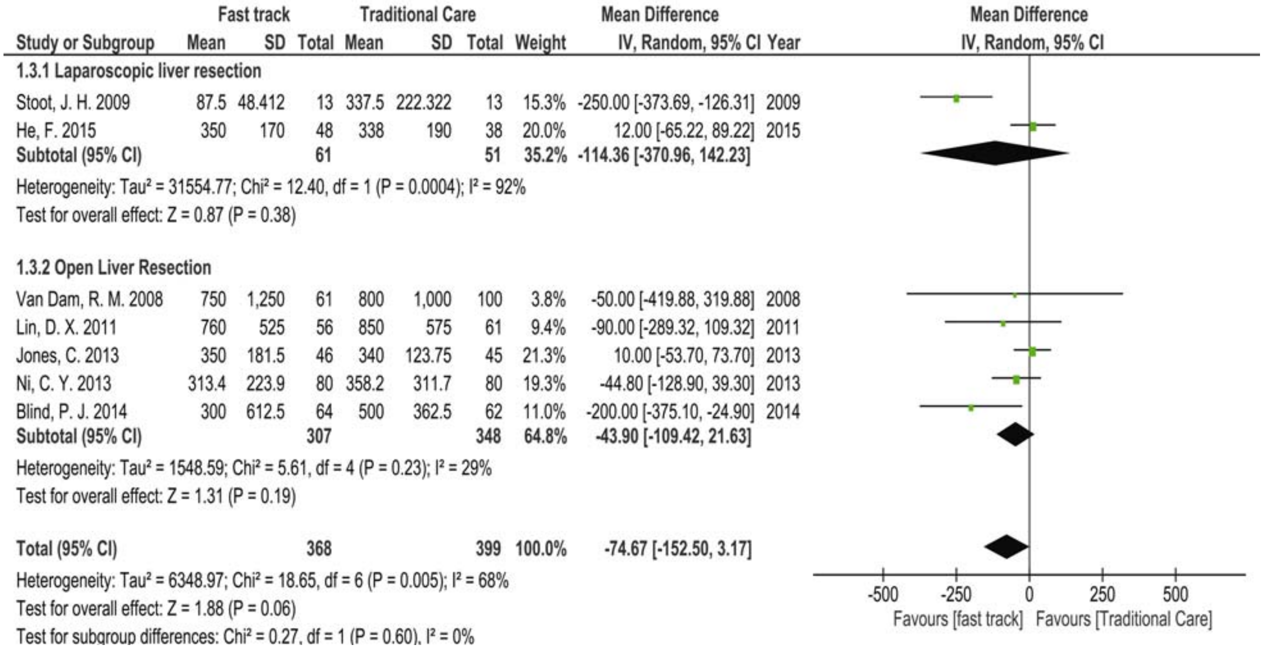

Supplemental Digital Content 3, Figure that illustrates the Forest plot of comparison: Fast Track vs. Traditional Care, outcome: need for blood transfusion.

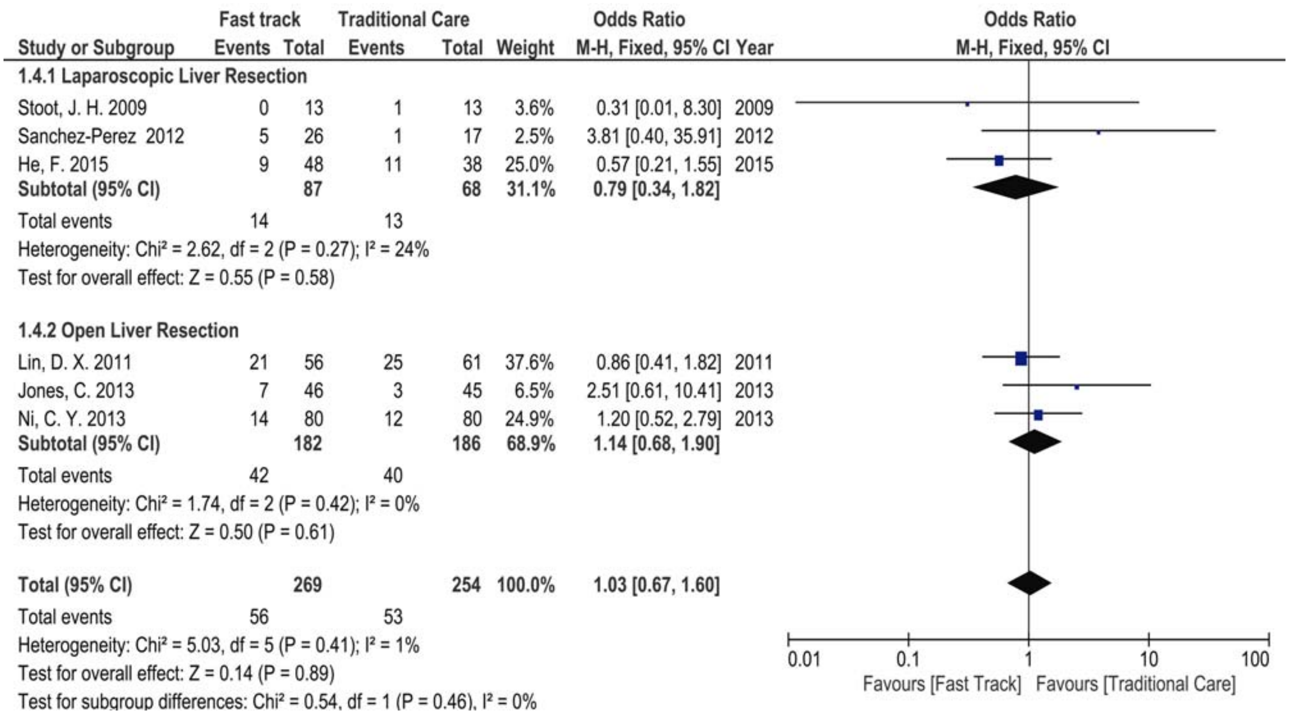

Supplemental Digital Content 4, Figure that illustrates the Forest plot of comparison: Fast Track vs. Traditional Care, outcome: conversion rate.

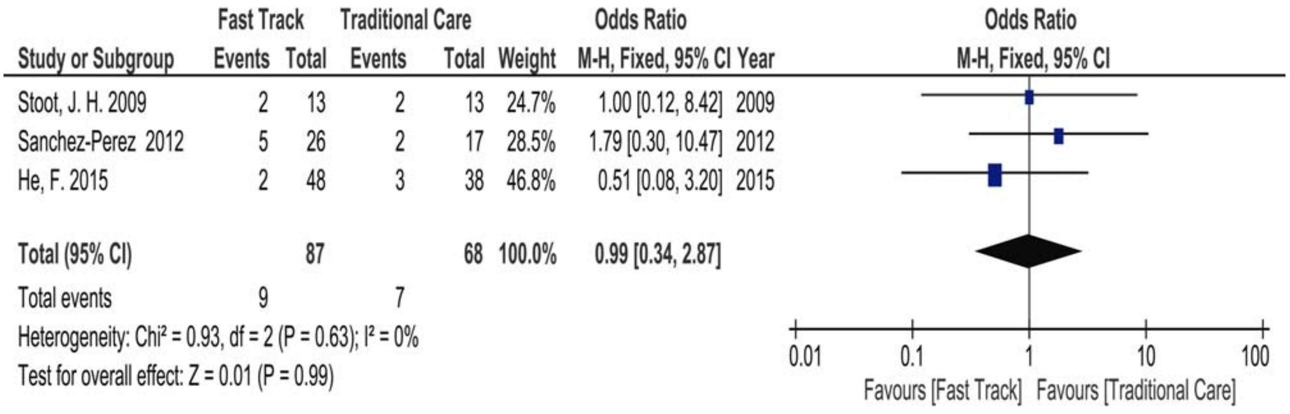

Supplemental Digital Content 5, Figure that illustrates the Forest plot of comparison: Fast Track vs. Traditional Care, outcome: mortality rate.

Study or Subgroup	Fast Track Events	Fast Track Total	Traditional Care Events	Traditional Care Total	Weight	Odds Ratio M-H, Fixed, 95% CI	Year
**1.18.1 Laparoscobic Liver Resection**							
Stoot, J. H. 2009	0	13	0	13		Not estimable	2009
Sanchez-Perez 2012	0	26	0	17		Not estimable	2012
He, F. 2015	0	48	0	38		Not estimable	2015
**Subtotal (95% CI)**		**87**		**68**		**Not estimable**	
Total events	0		0				
Heterogeneity: Not applicable							
Test for overall effect: Not applicable							
**1.18.2 Open Liver Resection**							
Van Dam, R. M. 2008	0	61	2	100	49.4%	0.32 [0.02, 6.78]	2008
Lin, D. X. 2011	1	56	1	61	24.6%	1.09 [0.07, 17.87]	2011
Ni, C. Y. 2013	0	80	0	80		Not estimable	2013
Jones, C. 2013	1	46	1	45	25.9%	0.98 [0.06, 16.12]	2013
Blind, P. J. 2014	0	64	0	0		Not estimable	2014
**Subtotal (95% CI)**		**307**		**286**	**100.0%**	**0.68 [0.14, 3.34]**	
Total events	2		4				
Heterogeneity: Chi² = 0.41, df = 2 (P = 0.82); I² = 0%							
Test for overall effect: Z = 0.47 (P = 0.64)							
**Total (95% CI)**		**394**		**354**	**100.0%**	**0.68 [0.14, 3.34]**	
Total events	2		4				
Heterogeneity: Chi² = 0.41, df = 2 (P = 0.82); I² = 0%							
Test for overall effect: Z = 0.47 (P = 0.64)							
Test for subgroup differences: Not applicable							

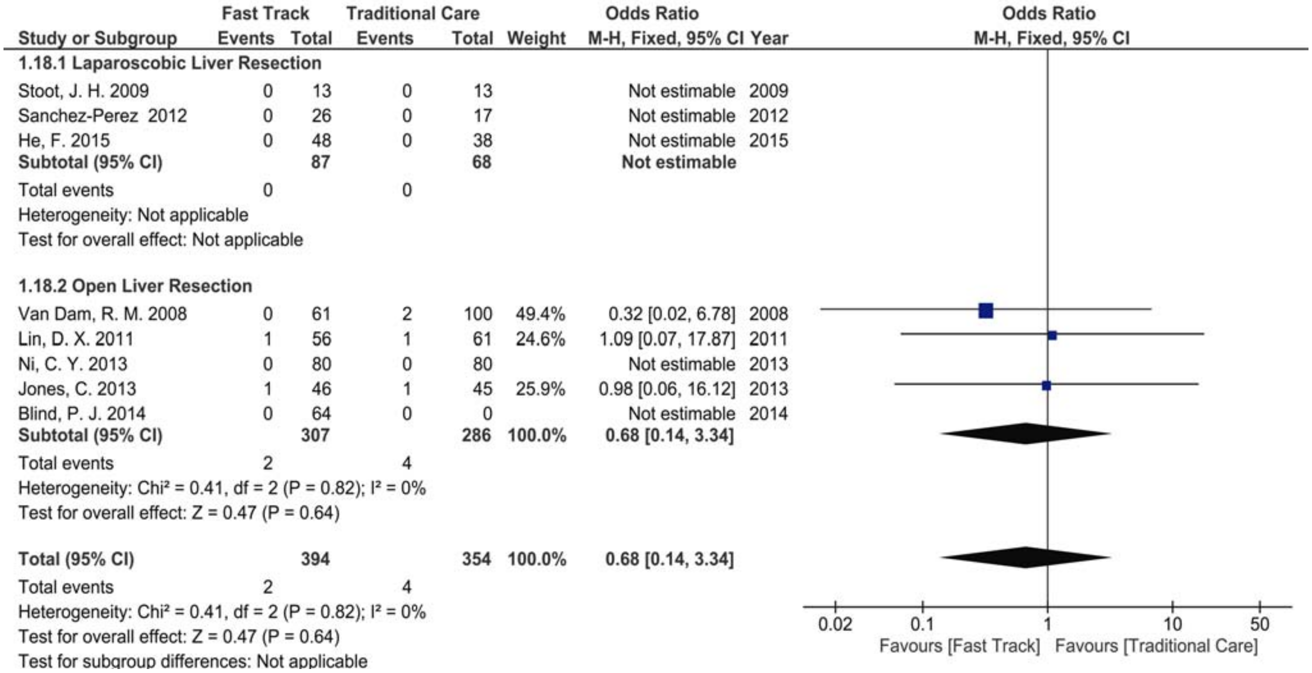

Supplemental Digital Content 6, Figure that illustrates the Forest plot of comparison: Fast Track vs. Traditional Care, outcome: readmission rate.

Study or Subgroup	Fast Track Events	Fast Track Total	Traditional Care Events	Traditional Care Total	Weight	Odds Ratio M-H, Fixed, 95% CI	Year
Van Dam, R. M. 2008	8	61	10	100	38.9%	1.36 [0.50, 3.65]	2008
Stoot, J. H. 2009	0	13	0	13		Not estimable	2009
Lin, D. X. 2011	4	56	2	61	10.5%	2.27 [0.40, 12.90]	2011
Sanchez-Perez 2012	1	26	1	17	6.9%	0.64 [0.04, 10.98]	2012
Jones, C. 2013	2	46	0	45	2.8%	5.11 [0.24, 109.51]	2013
Blind, P. J. 2014	3	64	6	62	34.4%	0.46 [0.11, 1.92]	2014
He, F. 2015	1	48	1	38	6.5%	0.79 [0.05, 13.01]	2015
**Total (95% CI)**		**314**		**336**	**100.0%**	**1.17 [0.61, 2.23]**	
Total events	19		20				
Heterogeneity: Chi² = 3.42, df = 5 (P = 0.64); I² = 0%							
Test for overall effect: Z = 0.46 (P = 0.64)							

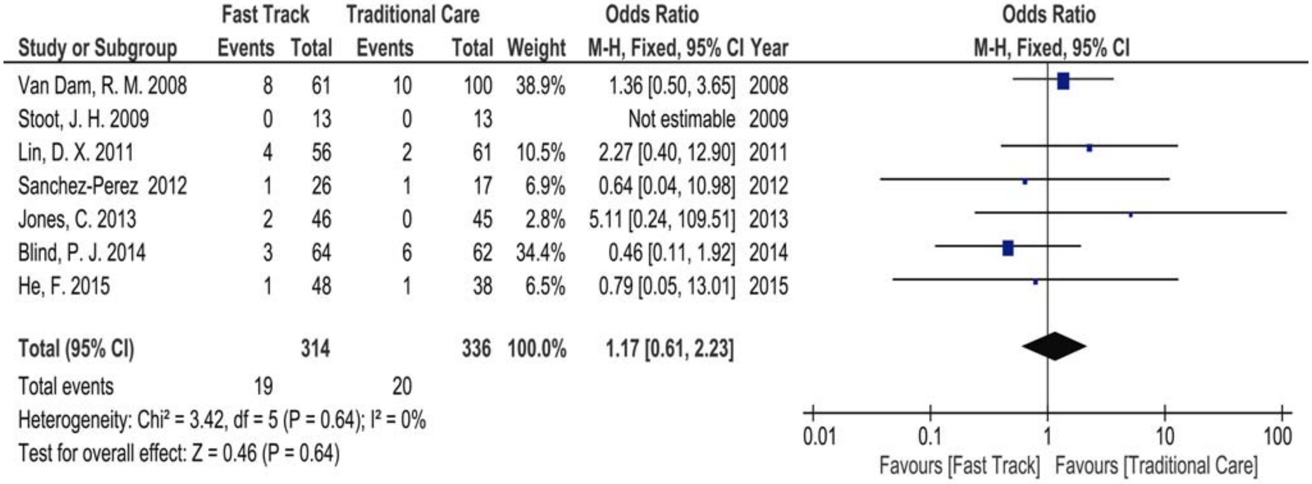

Table 1: Indications and extent of Liver Resection in each study.

	Van Dam, 2008		Stoot, 2009		Lin, 2011		Sanchez-P., 2012		Ni, 2013		Jones, 2013		Blind, 2014		He, 2015	
	**FT n=61**	**TC n=100**	**FT n=13**	**TC n=13**	**FT n=56**	**TC n=61**	**FT n=26**	**TC n=17**	**FT n=80**	**TC n=80**	**FT n=46**	**TC n=45**	**FT n=64**	**TC n=62**	**FT n=48**	**TC n =38**
Indications																
Colorectal Metastasis	51	72			NA	NA	8	0	0	0	35	26	53	42	20	16
Other Metastasis	2	4	5	2	NA	NA	-	-	-	-	-	-	-	-	-	-
Hepatocellular Carcinoma	4	10			NA	NA	3	3	71 9	76 4	0	0	6	17	11	8
Cholangiocarcinoma					NA	NA	1	0			0	0	0	0	0	0
Benign Lesions	4	14	8	11	NA	NA	14	14	0	0	1	9	5	3	17	14
Resection																
segmentectomy	10	21	3	3	13	14	0	0	7	11	25	33	NA	NA	5	2
Bisegmentectomy	0	0	9	9	17	17	5	4	0	0	0	0	NA	NA	43	36
Wedge Resection	0	0	0	0	7	9	12	7	0	0	0	0	NA	NA	0	0
Major Resection	51	79	1	1	19	21	0	0	73	69	21	12	22	26	0	0
Others	0	0	0	0	0	0	9	6	0	0	0	0	NA	NA	0	0

NA: Not Available.
